# A Dual-Reporter System for Real-Time Monitoring and High-throughput CRISPR/Cas9 Library Screening of the Hepatitis C Virus

**DOI:** 10.1038/srep08865

**Published:** 2015-03-09

**Authors:** Qingpeng Ren, Chan Li, Pengfei Yuan, Changzu Cai, Linqi Zhang, Guangxiang George Luo, Wensheng Wei

**Affiliations:** 1 Biodynamic Optical Imaging Center (BIOPIC), Peking-Tsinghua Center for Life Sciences, State Key Laboratory of Protein and Plant Gene Research, School of Life Sciences, Peking University, Beijing 100871, China; 2 Comprehensive AIDS Research Center and Research Center for Public Health, School of Medicine, Tsinghua University, Beijing 100084, China; 3 Department of Microbiology, University of Alabama at Birmingham School of Medicine, Birmingham, Alabama 35294, USA; 4 Department of Microbiology, Peking University College of Basic Medical Sciences, Beijing 100083, China

## Abstract

The hepatitis C virus (HCV) is one of the leading causes of chronic hepatitis, liver cirrhosis and hepatocellular carcinomas and infects approximately 170 million people worldwide. Although several reporter systems have been developed, many shortcomings limit their use in the assessment of HCV infections. Here, we report a real-time live-cell reporter, termed the NIrD (NS3-4A Inducible rtTA-mediated Dual-reporter) system, which provides an on-off switch specifically in response to an HCV infection. Using the NIrD system and a focused CRISPR/Cas9 library, we identified *CLDN1*, *OCLN* and *CD81* as essential genes for both the cell-free entry and the cell-to-cell transmission of HCV. The combination of this ultra-sensitive reporter system and the CRISPR knockout screening provides a powerful and high-throughput strategy for the identification of critical host components for HCV infections.

Since it was first identified in 1989^1^, HCV has become increasingly important in diseases associated with viral hepatitis, as well as HIV infections^2^,^3^. Although progress has been made in identifying key events that occur in host cells upon HCV infection over the past two decades^4^,^5^,^6^,^7^,^8^,^9^, the lack of a highly effective method to monitor the viral infection has hindered advances in the study of HCV. Specifically, it is difficult to distinguish between the two routes of HCV infection, cell-free entry and cell-to-cell transmission^10^,^11^,^12^,^13^,^14^. We aim to create a system that overcomes the various imperfections of the current methods of HCV monitoring^15^,^16^,^17^,^18^,^19^,^20^,^21^,^22^ and, consequently, to establish a high-throughput strategy to globally investigate the role of host genes in HCV infections.

The design of this novel HCV reporter consists of two modules, a sensor and an amplifier. The sensor is the chimeric protein rtTA-MAVS(C) (reverse tetracycline transactivator^23^,^24^ - mitochondrial antiviral signalling protein^25^ (C-terminal amino acids 462–540)) that contains the NS3-4A cleavage site. The amplifier is an expression module composed of the tight-TRE promoter^26^ followed by the coding sequences of 2A-linked delta-TK^27^ and mCherry (Fig. 1a). To minimise a potential leakage problem, the sensor and amplifier are spatially separated, with the former anchored to the cytoplasmic mitochondria and the latter located in the nucleus. In addition, the activation of the tight-TRE promoter requires both rtTA and doxycycline (Dox)/tetracycline, further minimising its non-specific activation. This live cell reporter, designated as the NIrD (NS3-4A Inducible rtTA-mediated Dual-reporter) system, provides an on-off switch that specifically responds to an HCV invasion. Upon inoculation, HCV-encoded NS3-4A protease cleaves rtTA-MAVS(C) in mitochondria, and the free-formed rtTA subsequently enters the nucleus, where it binds to and activates the tight-TRE promoter in the presence of Dox, resulting in de novo expression of delta-TK-2A-mCherry. After 2A-mediated cleavage, mCherry gives rise to red fluorescence and delta-TK leads to cell death in the presence of GCV (Ganciclovir)^27^ (Fig. 1a). All elements of the sensor and amplifier were combined into a single lentiviral backbone, pLenti-NIrD (Supplementary Fig. 1), making it convenient to acquire stable clones with the integrated NIrD system in any given cell type through viral infection and Blasticidin selection.

To examine the efficiency and specificity of this new reporter, we first established the NIrD system in a non-hepatic cell line, HeLa. Strong red fluorescence was observed in HeLa(NIrD) cells only when they were infected by Lenti(NS3-4A) and supplied with Dox (2 μg/ml). Interestingly, HCVcc (JFH-1 strain)^28^, unlike lentivirally expressed NS3-4A, failed to induce red fluorescence even in the presence of Dox, which is consistent with previous reports that non-hepatic cells lack key receptors that allow HCV entry^29^ (Supplementary Fig. 2a). Similarly, only the lentivirally expressed NS3-4A, not HCVcc, induced delta-TK-mediated death in the presence of GCV in HeLa cells (Supplementary Fig. 2b). The finding that Dox is required for all reporter gene expression demonstrates that reporter production is under the complete control of the tight-TRE promoter (Supplementary Fig. 2).

We then introduced the NIrD system to a hepatic cell line, Huh7.5, and selected Blasticidin-resistant clones for further analysis. Lentivirally delivered NS3-4A in the presence of Dox again induced red fluorescence (Supplementary Fig. 3a) and delta-TK-mediated death in the presence of GCV at 96 h post-infection (Supplementary Fig. 4). The red fluorescence peaked 72–96 h following the viral infection (Supplementary Fig. 3b). HCVcc inoculation induced mCherry fluorescence only in the presence of Dox, indicating that the NIrD system is capable of specifically detecting an HCV infection (Fig. 1b). The production of the 2^nd^ reporter delta-TK upon HCV infection (+Dox) led to cell death when GCV was supplied (Fig. 1c). These dual reporters, both responding specifically to an HCV challenge, provide additional flexibility for the use of this system.

We further confirmed that the mCherry fluorescence was tightly associated with HCV infection, where HCV viral particles were detected by anti-core antibody plus Alexa Fluor 488-conjugated secondary antibody (Fig. 1d). Consistently, red and green fluorescence coexisted when Huh7.5(NIrD) cells were infected by HCVcc encoding NS5A-EGFP^28^ (Supplementary Fig. 5). Immunoblotting analysis confirmed that Dox did not affect the viral infection as determined by the similar level of HCV core protein inside the cells (Fig. 1e). Based on our design, the transactivation of the dual reporters is mediated by NS3-4A activity, which cleaves and releases rtTA from the mitochondria (Fig. 1a). Indeed, the red fluorescent signals correlated inversely with the amount of VX-950 (i.e., Telaprevir), an NS3-4A inhibitor and newly marketed drug approved by FDA^30^. The finding that 500 nM of VX-950 blocked nearly all of the mCherry production indicates that the NIrD system is suitable for the screening of chemical compound inhibitors against HCV (Fig. 1f).

To quantitatively evaluate the NIrD system in response to HCV infections, we monitored the mCherry signal upon viral inoculation at different time points. The red fluorescence did not appear until 48 h after the infection, and the number of cells showing fluorescence increased over time (Fig. 2a). Uninfected cells showed no detectable signals in FACS analysis, while Huh7.5(NIrD) responded with high sensitivity to the viral infection; in total, 96.6% of infected Huh7.5(NIrD) cells displayed red fluorescence when the titre of HCVcc reached 2.51 × 10^4^ TCID50/ml (Fig. 2b). These results indicate a superior signal/noise ratio, a finding that was further confirmed by detailed FACS analysis, which showed that the reporter signals and HCV titres had a strong linear correlation (R^2^ = 0.995) within a dynamic range (Fig. 2c-d). In particular, the NIrD system was sensitive enough to detect HCV with a MOI as low as 0.001 (Fig. 2d).

Next, we investigated whether the NIrD system could be used for large-scale screening purposes. Taking advantage of a functional screening technique that we have recently developed^31^, we screened a focused CRISPR library in Huh7.5(NIrD) cells, in which OCT1-Cas9 expression was pre-established through lentiviral infection and neomycin selection (Fig. 3a). One particular clone, designated as Huh7.5(NIrd)_OC_-SC14, was selected to construct the library because it showed the highest level of efficiency in creating indels using *CSPG4*-targeting sgRNA^31^ and increased its efficiency in causing DNA double-strand breaks (DSBs) with prolonged culturing, as expected (Fig. 3b).

We used the same library as previously reported^31^, supplemented with additional sgRNAs, for a total of 918 sgRNAs that targeted 296 human genes, including all of the genes^14^ that have been reported to encode cell surface proteins important for HCV infections (Supplementary Table 1). Fifteen non-targeting sequences were also included in the library as negative controls. The CRISPR library built on Huh7.5(NIrD)_OC_-SC14 underwent 3–4 rounds of HCV challenges and FACS enrichment for those cells that no longer showed red fluorescence following the HCV inoculation (Fig. 3c). At the end of the last round of the HCV infection, over 99.4% of the cells in all three replications of the libraries showed no mCherry signal, while the majority of the control Huh7.5(NIrD)_OC_ cells remained responsive to HCV (Supplementary Fig. 6a). All Huh7.5(NIrD)_OC_ cells remained responsive to lentivirally delivered NS3-4A (Supplementary Fig. 6b), indicating that the NIrD system was functional even in the enriched Huh7.5(NIrD)_OC_ cells post-screening.

High-throughput sequencing analysis revealed a total of 912 sgRNA sequences (99.3% of the 918 designed) from the original library (Supplementary Table 2). We compared the abundance of each sgRNA between the final enriched Huh7.5(NIrD)_OC_ cells that no longer responded to HCV inoculation and the untreated populations and calculated a score for each sgRNA or gene using different algorithms (Fig. 4a–c). Three genes, *CLDN1*, *OCLN* and *CD81*, were confirmed to encode receptor proteins essential for HCV infections, based on false discovery rate (FDR) calculations^32^. It remains to be determined whether the other genes in the library are minimally involved in HCV infections or whether the number of designed sgRNAs targeting these genes were simply too small to produce data with statistical significance.

To further verify if these three proteins play an indispensible role in HCV infections as previously reported^4^,^6^,^7^, we created individual *CLDN1*, *OCLN* and *CD81* knockouts in Huh7.5(NIrD) cells using either the TALEN technique^33^ or the CRISPR/Cas9 system^31^. Clones in Huh7.5(NIrD) cells with the complete knockout of these genes were isolated, and Sanger sequencing analysis revealed that Huh7.5 (NIrD) *CLDN1*^−/−^ contained a 31-nt insertion in the TALEN-targeting region, Huh7.5(NIrD) *OCLN*^−/−^ contained a 17-nt insertion in the CRISPR sgRNA-targeting region, and Huh7.5(NIrD) *CD81*^−/−^ contained a 8-nt deletion in the CRISPR sgRNA-targeting region; all of these changes led to frame shifts (Supplementary Fig. 7a–c). The loss of the gene expression of any of these three genes completely blocked HCV infections, while the exogenous expression of their corresponding genes in the knockout lines rescued these lost phenotypes (Fig. 4d). These results are consistent with prior reports for the essential roles of CD81, CLDN1 and OCLN as receptors or co-receptors facilitating the HCV internalisation process^4^,^6^,^7^.

We next investigated role of these genes in the cell-to-cell transmission of HCV, another important route for the spread of the virus^34^,^35^. Huh7.5(NIrD) cells were mixed with donor Huh7.5 cells pre-loaded with EGFP-labelled HCVcc^28^, and red fluorescence appeared after 72 h (+Dox), indicating that HCV was transmitted through tight junctions to Huh7.5(NIrD) cells. Interestingly, this cell-to-cell transmission route was blocked in all three gene-knockout clones, while the expression of their corresponding genes restored the lost phenotypes (Fig. 4e). These results are consistent with previous findings that CLDN1 and OCLN are tight junction proteins critical for the cell-to-cell transmission of HCV^11^,^34^. The role of CD81 in the spread of the virus between cells is somewhat controversial^10^,^11^,^12^, and our results support its active role in HCV transmission between neighbouring cells.

Current HCV reporter systems are either viral-based or host cell-based. The viral-based strategy uses modified HCVcc or replicons of different subtypes to express reporters such as EGFP^36^,^37^ or luciferase^38^. However, the modification of HCV genome may be cumbersome, as the productivity of an artificial HCV may drop over time and the modified genome may create additional RNA structure deviations^37^. In addition, viral-based reporters are unable to distinguish between cell-free entry and cell-to-cell transmission routes for HCV. In comparison, cell-based reporter systems take advantage of the bio-activity of HCV-encoded proteins, such as NS3-4A protease^18^,^39^,^40^,^41^,^42^ and NS5B RNA polymerase^43^. The NIrD system we report here uses multiple layers of regulations, including a sensor and an amplifier, to ensure minimal background noise and maximal signals through amplification specifically in response to HCV inoculation. The NIrD system is particularly advantageous in the study of HCV transmission, as it does not require complex processes such as antibody staining^8^. The applicability of the NIrD system to a broad range of HCV genotypes is also supported by the finding that the NS3-4A cutting site is sensitive to numerous HCV genotypes^18^,^44^. Nevertheless, since NIrD system was solely dependent on the functional production of NS3/4A protease, this reporter is only useful for detecting early events of HCV life cycle.

In summary, we developed a cell-based dual-reporter system that demonstrates a superior ability to monitor an HCV infection in real-time. In addition to the outstanding signal/noise ratio, the NIrD system provides on-off switching signals upon HCV infection, which makes it an ideal system for the high-throughput study of HCV infections either through FACS or suicidal selection. In addition, the two reporters in the NIrD system could be substituted for other reporter types, such as luciferase^38^ or SEAP^39^,^42^, depending on specific needs. Importantly, the NIrD system is well-suited for the study of viral transmission processes, a difficult task for the majority of other reporter types^28^,^36^,^37^. Although the NIrD system is unlikely applicable for monitoring primary viruses (Supplementary Fig. 8) because these clinical strains lack the replication capability in cell culture condition, it has shown unique advantages in studies of both HCV cell-free entry and cell-to-cell transmission. In combination with current gene editing tools^31^,^45^,^46^,^47^, NIrD is a robust and powerful system for the study of complex host responses to HCV, particularly in a high-throughput fashion based on a CRISPR/Cas9 library screening. A more thorough investigation of the NIrD system that uses a library that targets all protein-encoding genes is highly desirable and is highly likely to reveal comprehensive information regarding host mechanisms in response to HCV infections.

## Methods

### Cell culture

Huh7.5 cells were cultured in Dulbecco's Modified Eagle Medium (DMEM, Life Technologies) containing 10% FBS (Thermo Scientific), 1× penicillin-streptomycin and 1× nonessential amino acids (NEAA, Life Technologies) at 37°C and 5% CO_2_. For the induction of the reporter expression, culture media was supplemented with either 2 μg/ml Dox or 2 μg/ml Dox + 2 μg/ml GCV. HeLa and 293FT cells were cultured in DMEM containing 10% FBS.

### Plasmid construction

The pLenti-NIrD plasmid was constructed through standard cloning methods, and the sequences of the primers and plasmids are available upon request. All modules were inserted into a lentiviral vector, pLentiCMVMCSSVBsd. The CMV promoter was replaced with the tight-TRE promoter (from pEN_TTmcs), and DNA fragments coding delta-TK (a modified version of HSV-TK), 2A (2A of the Thosea asigna virus) and mCherry were concatenated sequentially. rtTA-MAVS(C)-IRES was inserted between the SV40 promoter and the Blasticidin marker gene. rtTA was cloned from pSLIK-Neo-TGmiR-Luc, and MAVS(C) (residues 462–540 of MAVS) was PCR-amplified from HeLa cDNA. The IRES sequence was obtained from pcDNA6HASIRESPuro. The TALENs plasmids were constructed using the ULtiMATE protocol^33^ developed in our lab. pLenti-OC-IRES-Neo was modified from the pLenti-OC-IRES-BSD plasmid^31^. CLDN1, CD81 and OCLN expression plasmids were constructed on pLentiCMVPuroDEST, pMSCVpuro and pLentiCMVMCSSVNeo, respectively.

### Construction of the CRISPR/Cas9 sgRNA library

We constructed a focused CRISPR/Cas9 library based on a previously reported library^31^ with the addition of 52 sgRNAs that targeted 9 genes and included 15 non-targeting sgRNAs as negative controls. This library consisted of 918 sgRNAs, targeting a total of 296 genes.

### CRISPR/Cas9 sgRNA library screening and candidate sgRNA identification

In total, 4 × 10^6^ library cells were seeded per 150 mm petri dish as one replicate, and a total of three replicates were used for screening. After 24 h, library cells were infected by HCVcc^48^ (26,666 TCID50/ml) in the presence of Dox (2 μg/ml). The original Huh(NIrD)_OC_-SC14 cells were also challenged with HCV as controls. The medium was changed to fresh DMEM 24 h post infection, and the cells were harvested in another 48 h for FACS analysis to collect those cells that were mCherry negative. Cells were cultured with 2 μg/ml Dox throughout the treatment. This process was repeated 3–4 times until greater than 99% of the library cells showed no red fluorescence upon HCVcc inoculation. The high-throughput sequencing analysis and identification of the sgRNAs from the 3 replicates and the untreated libraries was conducted following the same protocol as previously reported^31^.

### Statistical analysis

The computational program^32^ developed in the lab of Dr. Xiaole Liu at Harvard was used to perform the statistical analysis. We built a negative binomial model to calculate FDR values, and positively selected genes were those with FDR values less than 0.05.

### FACS analysis

Prior to FACS analysis, Huh7.5(NIrD)_OC_ cells were infected with various concentrations of HCVcc with a fixed Dox level of 2 μg/ml for 72 h. Then, cells were washed by PBS three times and harvested using 0.005% trypsin. Harvested cells were washed twice with PBS and once with FACS buffer (PBS/3% FBS) prior to analysis using a BD FACSAria™ III flow cytometer. FACS data analysis was performed using BD FACSDiva™ software.

### Cell-to-cell transmission of HCV

Wild-type Huh7.5 cells were infected by EGFP-labelled HCVcc^28^ for 24 h and then co-cultured with Huh7.5(NIrD) cells with a different background (WT, *CLDN1*^−/−^, *CLDN1*^−/−^/CLDN, *OCLN*^−/−^, *OCLN*^−/−^/OCLN, *CD81*^−/−^ or *CD81*^−/−^/CD81). After 72 h of co-culturing, the cells were washed with PBS 2 times prior to fluorescence microscopy analysis.

### Immunofluorescence microscopy

Huh7.5(NIrD) cells (2 × 10^5^ per well) were grown in 6-well plate, with a coverslip in each well. Cells were infected by HCVcc^48^ with low MOI in the presence of Dox (2 μg/ml) for 72 h before fixed with PBS containing 4% methanol overnight at 4°C. Mouse anti-core mAb (1:100, Abcam #ab2740) and Alexa Fluor 488 goat anti-mouse IgG (H + L) antibody (1:1000, Life Technologies A-11029) were sequentially incubated with the sample for 1 h at 37°C, followed by PBS washing. Images were taken by DeltaVision Elite (Applied Precision, Issaquah, WA). Detailed protocol is the same as previously described^49^.

### Preparation and titration of HCVcc

Seed Huh7.5 cells (4 × 10^6^/150-mm dish) one day before HCVcc infection. The virus was harvested from the supernatant through centrifugation after 4 days of initial viral infection. The virus was stocked at −40°C. HCVcc titration was performed by seeding Huh7.5(NIrD) cells (1 × 10^4^ cells/well) in 96-well plates. Virus stock was serially diluted (10×) in DMEM medium prior to infection (100 μl of diluted virus/well, and typically 6 wells per dilution). The titre was calculated using Reed-Muench method^50^,^51^. MOI was calculated according to the number of particles of HCVcc (TCID50) divided by number of seeded cells.

## Author Contributions

W.W., Q.R. and C.L. conceived the methodology and designed the experiments. Q.R., C.L. and C.C. performed the experiments. W.W., Q.R., C.L., P.Y. and C.C. analysed the data. L.Z. and G.L. provided the reagents and the facility. W.W., Q.R. and C.L. wrote the manuscript with the assistance of the other authors.

## Supplementary Material

Supplementary Information Supplementary information

Supplementary Information Supplementary table 1

Supplementary Information Supplementary table 2

## Figures and Tables

**Figure 1:**
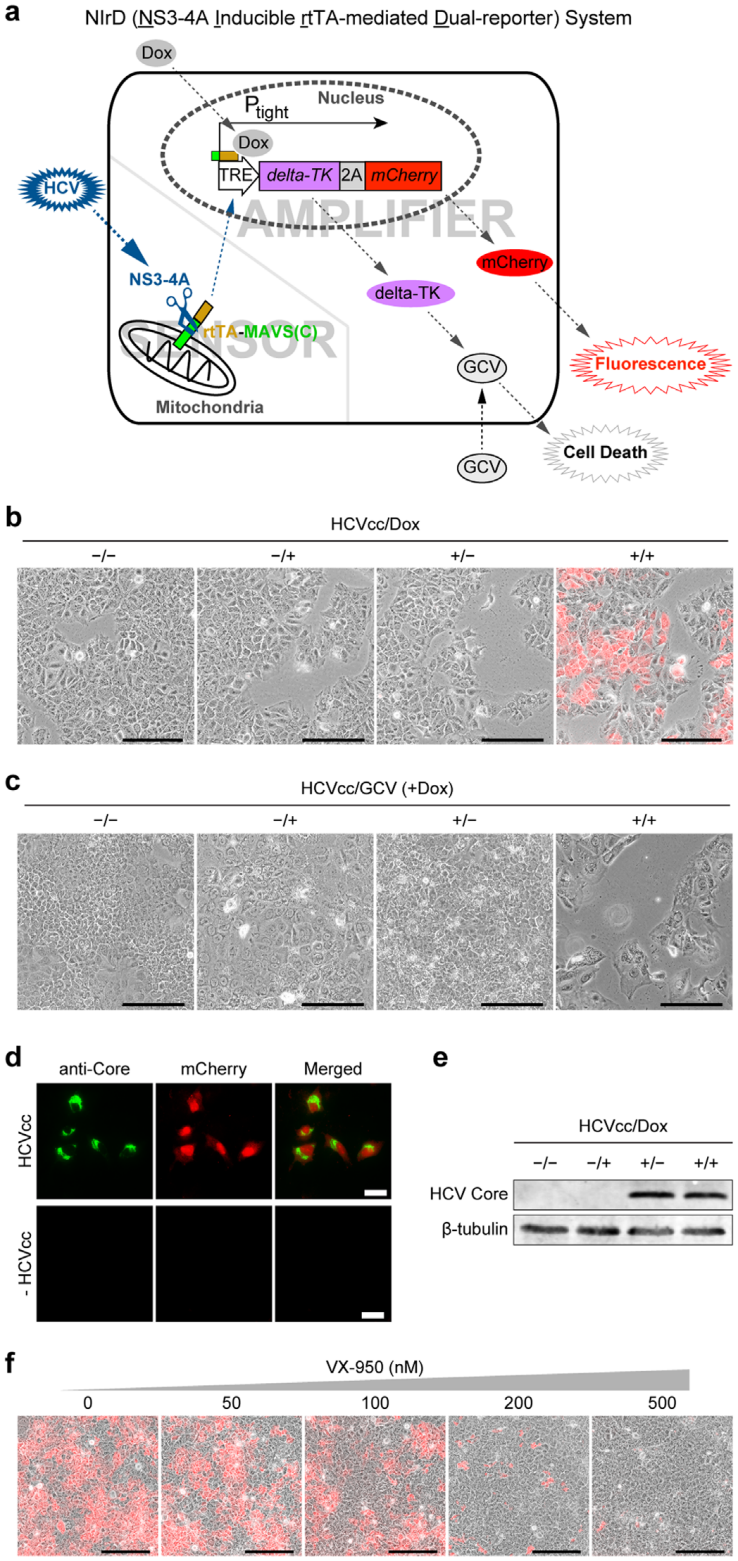
A cell-based dual-reporter system for monitoring HCV infections. (a) The rationale of the NIrD system. The sensor module consists of rtTA-MAVS(C) fusion proteins, which are constantly produced and localised to the mitochondria in cells. The amplifier module is an expression cartridge integrated into the chromosome that is composed of two reporter genes driven by the tight-TRE promoter. Upon HCV infection, the virally produced NS3-4A cleaves its recognition sequence in MAVS(C), releasing the free-formed rtTA into the nucleus. The tight-TRE promoter is activated by rtTA and Dox (2 μg/ml), resulting in the production of delta-TK-2A-mCherry proteins. After self-cleavage of the 2A peptide, mCherry shows red fluorescence and delta-TK phosphorylates GCV (2 μg/ml) to cause cell death. (b) mCherry signal of the NIrD system in response to an HCVcc infection. The Huh7.5(NIrD) system was infected by HCVcc for 72 h. HCVcc-transduced (+) or -untransduced (−) cells in the presence (+) or absence (−) of Dox (2 μg/ml) were visualised under a microscope. The fluorescence signals were superimposed onto white light images. Scale bar, 200 μm. (c) The death signal of the NIrD system in response to an HCVcc infection in the presence of GCV (2 μg/ml). Huh7.5(NIrD) cells were infected with HCVcc for 120 h. The transduced (+) or untransduced (−) cells in the presence (+) or absence (−) of Dox (2 μg/ml) were visualised under a light microscope. Scale bar, 200 μm. (d) Fluorescence microscopy of HCVcc core protein (Alexa Fluor 488) and mCherry signal upon HCVcc infection (72 h) in Huh7.5(NIrD) cells. Scale bar, 30 μm. (e) Immunoblotting analysis of Huh7.5(NIrD) cells infected with or without HCVcc in the presence or absence of Dox (2 μg/ml). HCVcc was detected by an antibody specifically targeting the viral core protein, and β-tubulin was used as the loading control. (f) Dosage effects of VX-950 on the live-cell imaging of HCVcc-infected Huh7.5(NIrD) cells. Huh7.5(NIrD) cells were infected with HCVcc plus Dox (2 μg/ml) together with serially increasing dosages of VX-950 (Telaprevir). Fluorescence microscopic images were taken 72 h following the HCVcc infection. Scale bar, 200 μm.

**Figure 2:**
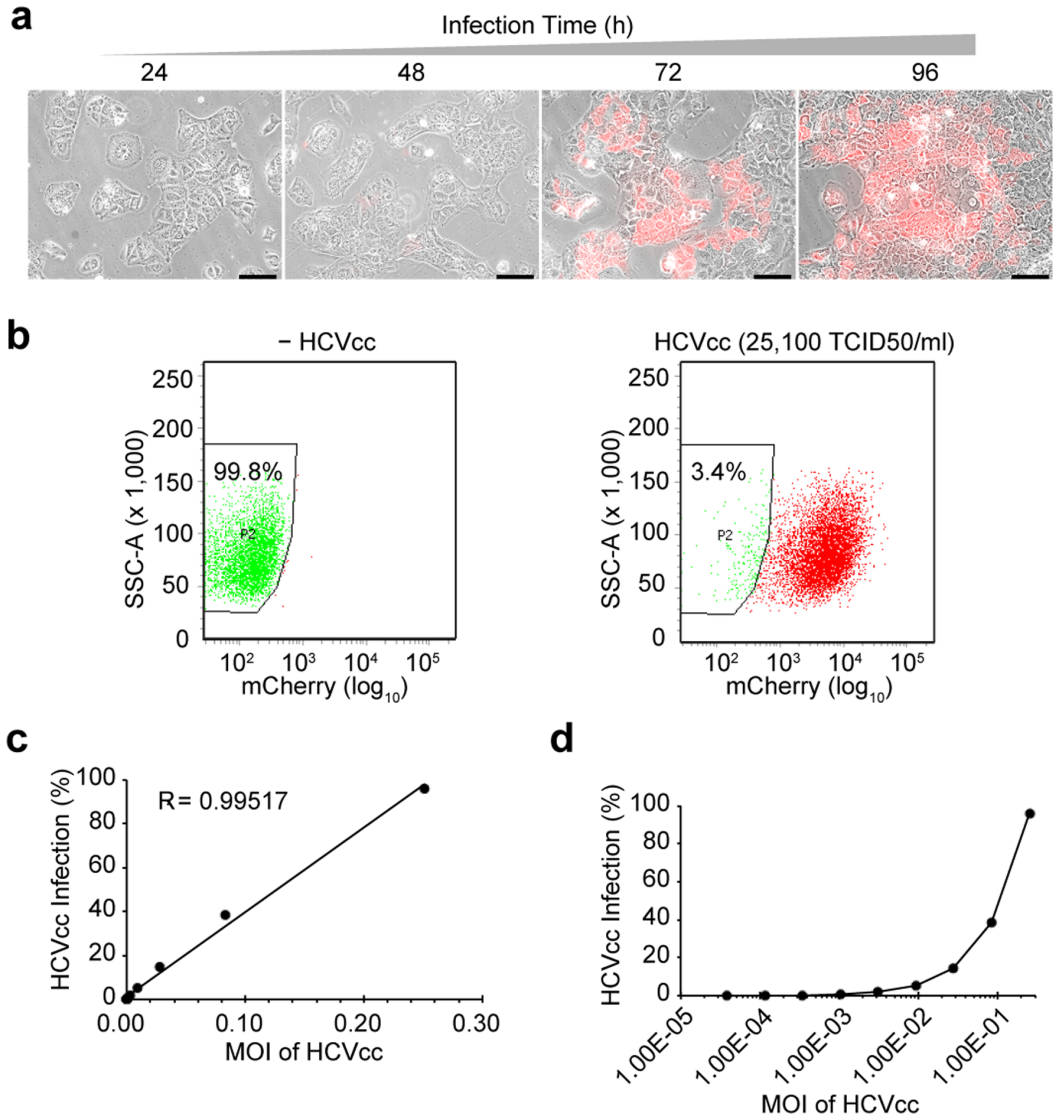
Quantitative evaluation of NIrD system in response to HCV inoculation. (a) Time-lapse live-cell imaging of Huh7.5(NIrD) cells. Both light and fluorescence images were taken every 24 h, starting at 24 h post-HCVcc infection in the presence of Dox (2 μg/ml). Scale bar, 100 μm. (b) FACS analysis of Huh7.5(NIrD) cells infected by HCVcc in the presence of Dox (2 μg/ml). A total of 2 × 10^5^/well of Huh7.5(NIrD) cells were seeded in 6-well plates. Representative results from reporter cells treated with HCVcc (0 or 25,100 TCID50/ml) are presented. FACS analysis was conducted 96 h following the viral infection. The numbers in the square indicate the percentage of red fluorescence-negative cells. (c-d) FACS analysis of Huh7.5(NIrD) cells infected by serially increasing dosages of HCVcc. The curves show the percentage of mCherry positive cells corresponding to MOI (2 × 10^5^ cells/well) in linear (c) or logarithmic (d) plots.

**Figure 3:**
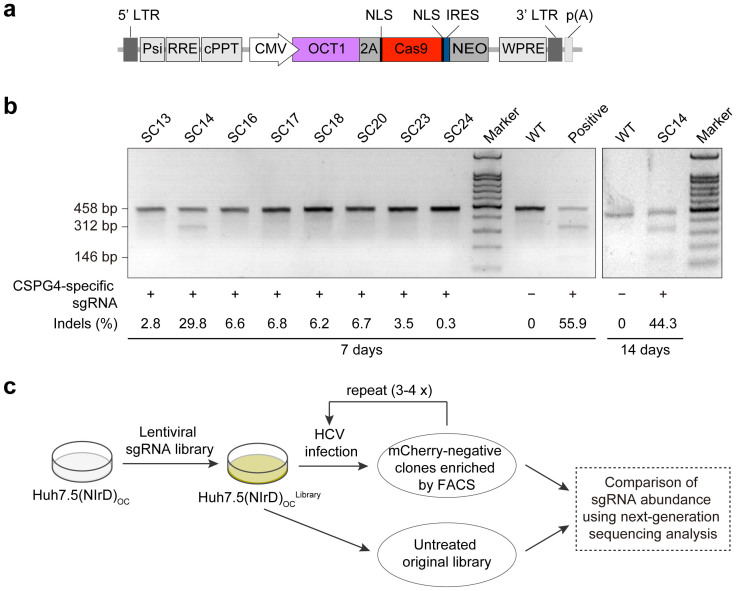
Schematic of the CRISPR library construction and HCV screening. (a) The structure of the lentiviral plasmid expressing OCT1 and Cas9. (b) Indels induced by the lentivirus-delivered sgRNA (5-TTGGCCAGACTTGCATCCG-3) targeting the *CSPG4* gene in the indicated cells were assayed by T7E1 digestion. Genomic DNA from HeLa_OC_-SC^31^ was used as a positive control, and the wild type (WT) Huh7.5(NIrD) was used as a negative control. (c) Schematic of the sgRNA library screening. sgRNAs were delivered into Huh7.5(NIrD)_OC_-SC cells by lentiviral infection with a MOI of 0.1. Three replicates of the libraries were challenged with 3–4 rounds of HCVcc, followed by FACS sorting to enrich the mCherry-negative clones. A comparison of the abundance of sgRNAs between the treated and untreated populations through high-throughput sequencing analysis was conducted following the same procedure as previously reported^31^.

**Figure 4:**
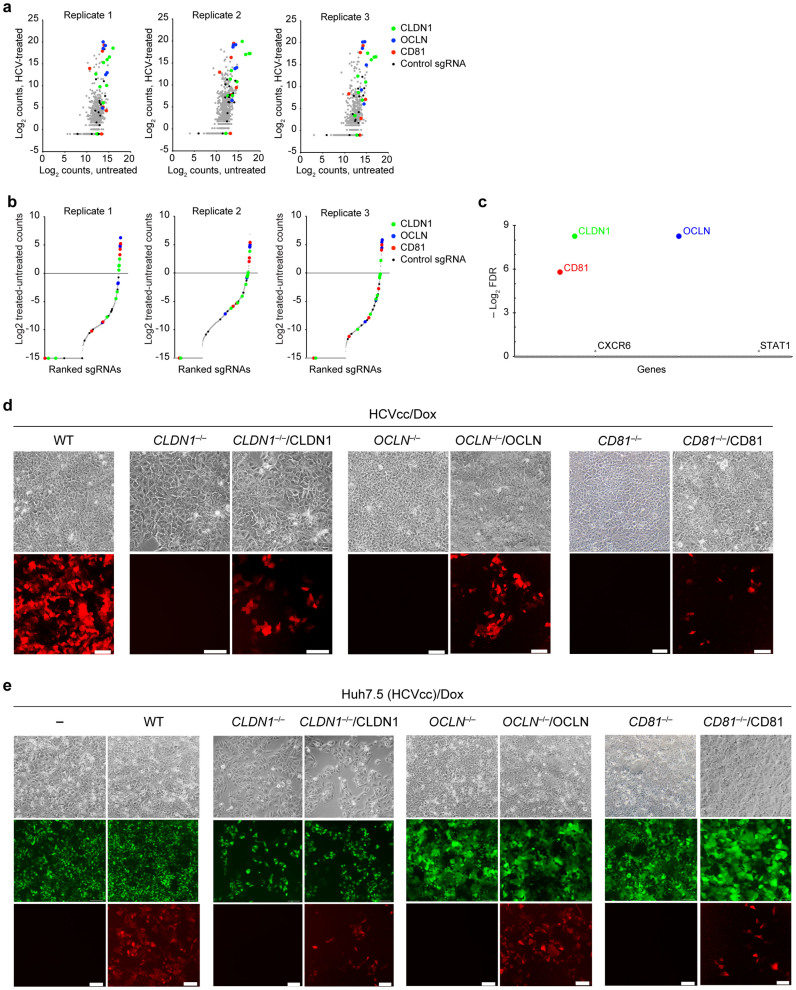
Screen of host genes essential for an HCV infection. (a) Primary HCVcc screening data. The count of every sgRNA is the number of reads that match the sgRNA target sequence. (b) sgRNA ranking was based on the fold change of normalised counts of every sgRNA in the HCV treated and untreated populations. (c) The FDR (false discovery rate) of every gene in the library was calculated by MAGeCK^32^ based on the counts and kinds of sgRNAs in the three replicates. (d) Effects of the gene knockout of CLND1, OCLN and CD81 on cell-free entry of HCVcc. All cells indicated carry the NIrD system. Light and fluorescence images were taken 72 h post-HCVcc infection in the presence of Dox (2 μg/ml). Scale bar, 100 μm. (e) Effects of the gene knockout of CLND1, OCLN and CD81 on cell-to-cell transmission of HCVcc. Huh7.5(NΙrD) cells with the indicated background (WT, *CLDN1*^−/−^, *CLDN1*^−/−^/CLDN1, *OCLN*^−/−^, *OCLN*^−/−^/OCLN, *CD81*^−/−^ or *CD81*^−/−^/CD81) were co-cultured with HCVcc pre-infected (24 h prior) Huh7.5 cells. HCVcc carries the EGFP gene in its genome^28^, resulting in a punctuated green fluorescence pattern in the cells. *OCLN*^−/−^ and *CD81*^−/−^ knockout cells expressed diffused green fluorescence because they were derived from cells expressing EGFP. The light and fluorescence (green and red) images were taken 72 h following the co-culturing of the cells. Scale bar, 100 μm.
