# Supplementary material for: A Dual-Reporter System for Real-Time Monitoring and High-throughput CRISPR/Cas9 Library Screening of the Hepatitis C Virus
Source: Sci Rep. 2015 Mar 9;5:8865. doi: 10.1038/srep08865 (PMC4352851; doi:10.1038/srep08865)
Supplement: Supplementary Information [file srep08865-s1.pdf]

Supplementary Information

A Dual-Reporter System for Real-Time Monitoring and High-throughput CRISPR/Cas9 Library Screening of the Hepatitis C Virus

Qingpeng Ren, Chan Li, Pengfei Yuan, Changzu Cai, Linqi Zhang, Guangxiang George Luo and Wensheng Wei

Biodynamic Optical Imaging Center (BIOPIC), State Key Laboratory of Protein and Plant Gene Research, School of Life Sciences, Peking University, Beijing 100871, China

*Correspondence should be addressed to W.W. (wswei@pku.edu.cn).

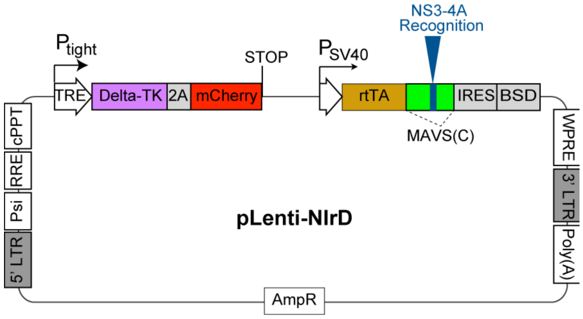

Supplementary Figure 1. Design of the NlrD construct. This reporter construct was composed of two cassettes in a pLentiCMVMCSSVBsd backbone. One cassette contains DNA sequences encoding 2A-linked delta-TK and mCherry under the control of the tight-TRE promoter (P_{tight}). The second cassette consists of the SV40 promoter-driven sequences encoding the rtTA-MAVS(C) fusion protein and Blasticidin (BSD), in which the rtTA were fused with MAVS C-terminal residues (462-540) that contain the NS3-4A recognition site (^{503}EREVPCH^{509}), and the BSD-coding region followed IRES.

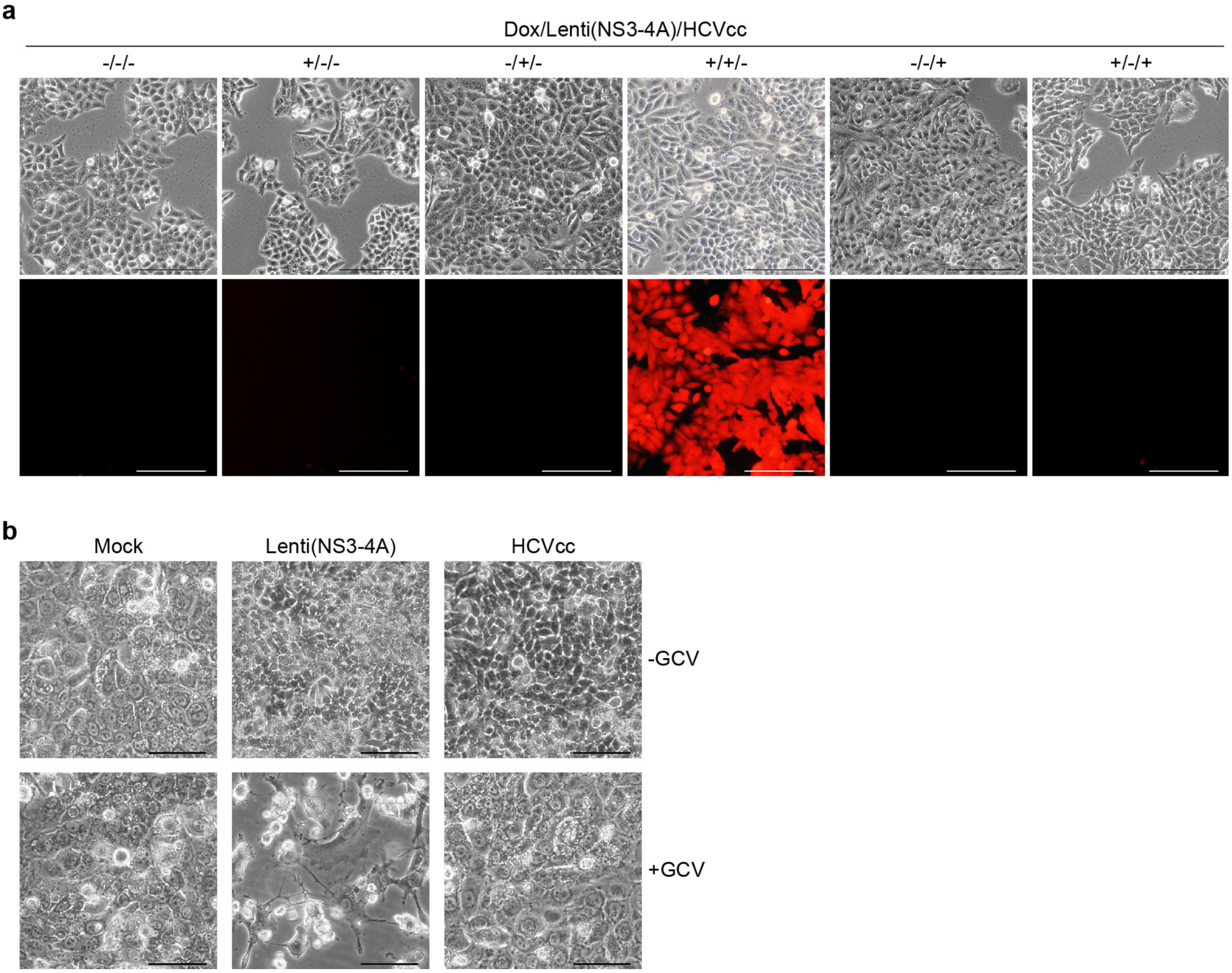

Supplementary Figure 2. The NlrD system in HeLa cells in response to NS3-4A-expressing lentivirus or HCVcc. (**a**) mCherry signal of the NlrD system in response to a viral infection. Both the light (top) and the fluorescent (bottom) images were taken 72 h post-infection with different combinations of Lenti(NS3-4A), HCVcc and Dox (2 μg/ml). Scale bar, 200 μm. (**b**) Death signal of the NlrD system in response to lentivirally delivered NS3-4A in the presence of Dox (2 μg/ml). The light images of the cells were taken 120 h post-infection in the absence (top) or presence (bottom) of GCV (2 μg/ml). Scale bar, 200 μm.

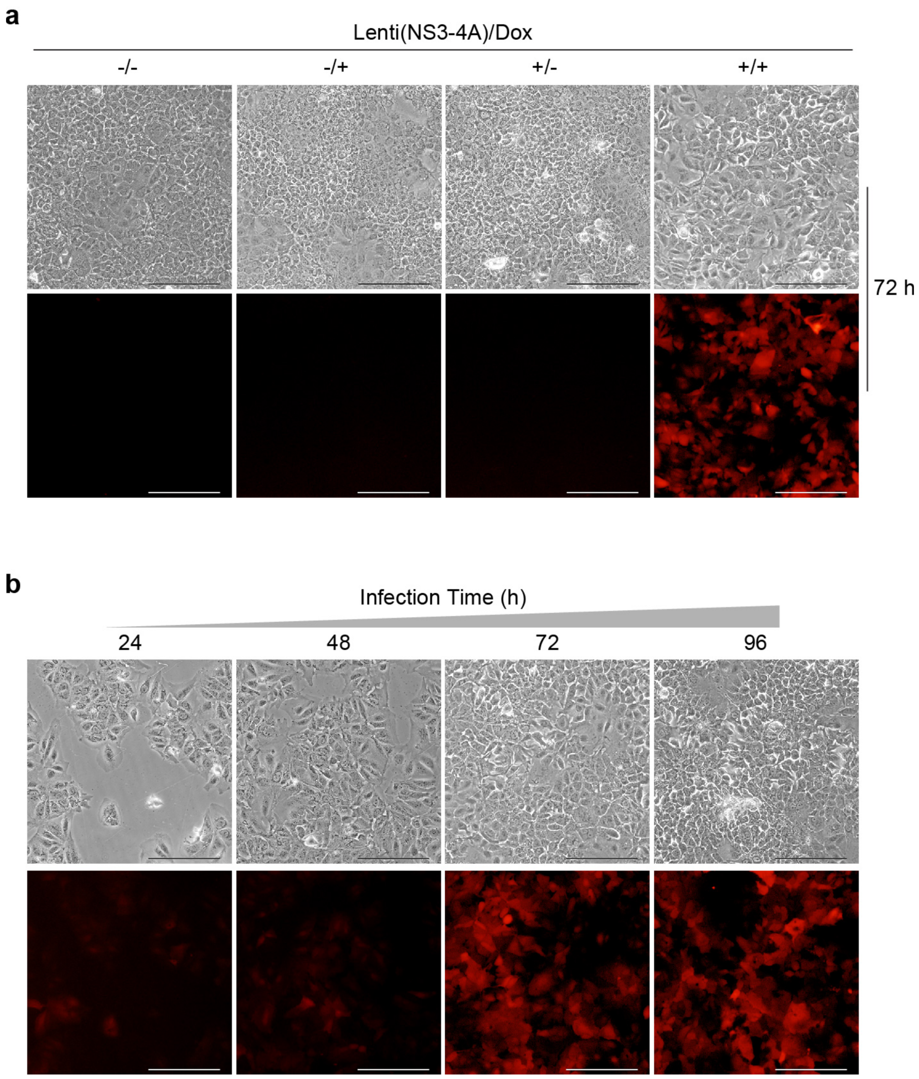

Supplementary Figure 3. The mCherry signal of the NIrD system in Huh7.5 cells in response to NS3-4A-expressing lentivirus. (**a**) Huh7.5(NIrD) cells were infected with the virus for 72 h. The virus-transduced (+) or -untransduced (−) cells in the presence (+) or absence (−) of Dox (2 μg/ml) were visualised by microscopy. Scale bar, 200 μm. (**b**) Time-lapse live-cell imaging of Huh7.5(NIrD) after NS3-4A-expressing lentiviral infection. Scale bar, 200 μm.

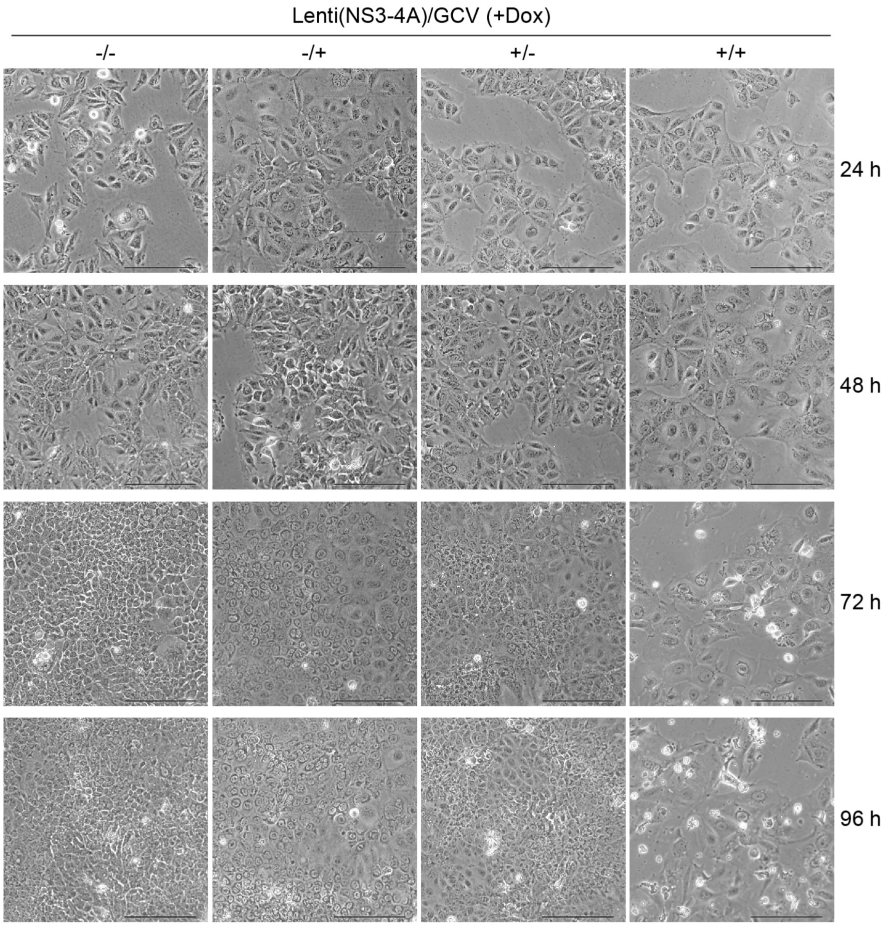

Supplementary Figure 4. Death signal of the NIrD system in Huh7.5 cells in response to NS3-4A-expressing lentivirus. Huh7.5(NIrD) cells were infected with (+) or without (−) the virus in the presence (+) or absence (−) of GCV (2 μg/ml). Dox (2 μg/ml) was supplied in all conditions. The light microscopy images were taken every 24 h, starting at 24 h post-infection. Scale bar, 200 μm.

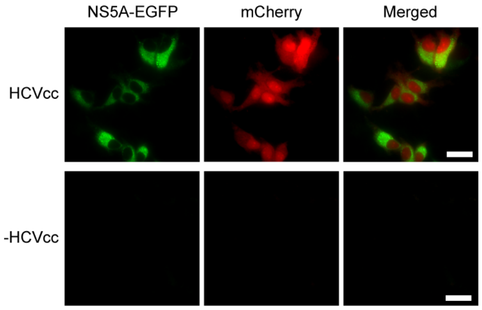

Supplementary Figure 5. Fluorescence microscopy of EGFP-conjugated NS5A and mCherry signal upon HCVcc infection (72 h) in Huh7.5(NIrD) cells. Scale bar, 30 μm.

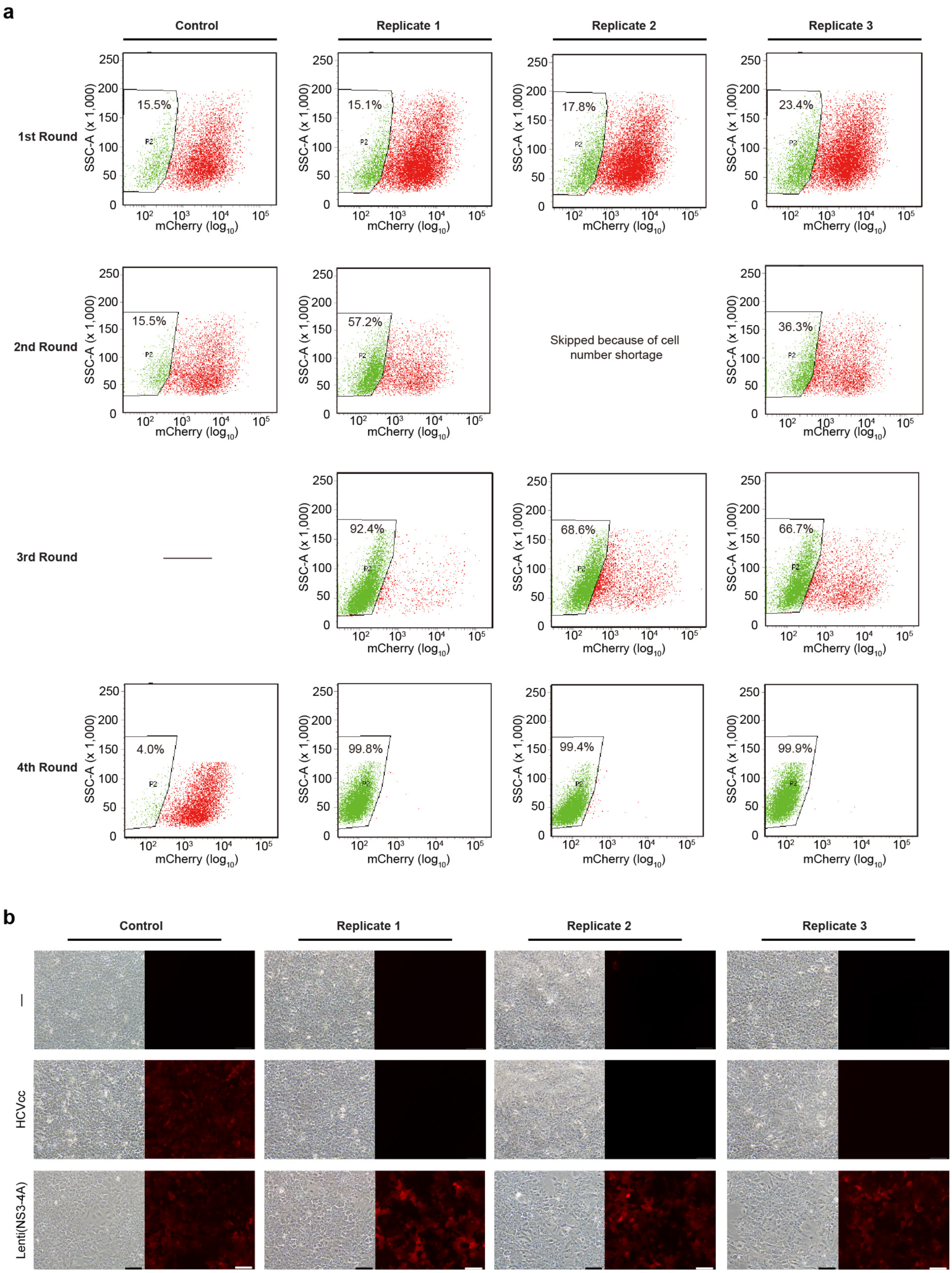

a
Control
Replicate 1
Replicate 2
Replicate 3
1st Round
2nd Round
3rd Round
4th Round
15.5%
15.1%
17.8%
23.4%
15.5%
57.2%
Skipped because of cell number shortage
36.3%
92.4%
68.6%
66.7%
4.0%
99.8%
99.4%
99.9%
P2
SSC-A (x 1,000)
mCherry (log_{10})
b
Control
Replicate 1
Replicate 2
Replicate 3
HCVcc
Lenti(NS3-4A)

Supplementary Figure 6. mCherry signals in Huh7.5(NIrD) cells during the library screening. (**a**) FACS analysis of control Huh7.5(NIrD) cells and three replicates of the CRISPR libraries throughout 3-4 rounds of HCVcc infections. In each round, 4×10^6 cells were infected by HCVcc (26,666 TCID50/ml) plus Dox (2 μg/ml), followed by FACS at 72 h post-viral infection. The original Huh7.5(NIrD) was used as the control for this and the remaining figures. (**b**) mCherry-negative cells in three library replicates following the 4th round of FACS were assayed through fluorescence microscopy on their internal NIrD system in response to NS3-4A-expressing lentivirus or HCVcc. Scale bar, 100 μm.

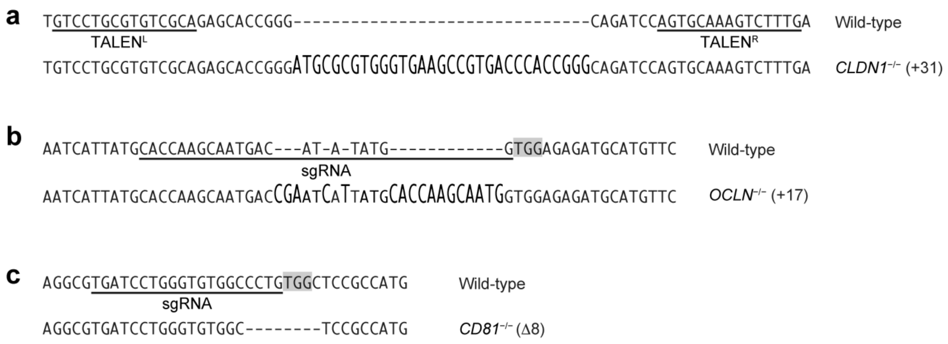

Supplementary Figure 7. Gene knockouts of CLDN1, OCLN and CD81 in Huh7.5(NIrD). (**a**) Partial sequences of the *CLDN1* gene in a genome containing TALENs binding regions (underlined) and the mutated alleles from the TALENs clone. The tall letter indicates an insertion and the dashes indicate a deletion in this and other figures. The primers used to amplify the TALENs targeting region for sequencing analysis are 5'-GTTGCCCACCTGCAAACTCTCC-3' and 5'-TGTCACCCAGGGAGTTAGAGGC-3'. (**b-c**) DNA sequencing analysis of mutated alleles in OCLN (**b**) and CD81 (**c**) loci. Partial coding sequences of the targeted genes in the genome containing the CRISPR/Cas9 sgRNA^{OCLN} or sgRNA^{CD81} binding regions (underlined) and the sequence analysis of the mutated alleles are shown. The shaded nucleotides represent the PAM sequences that guide Cas9 for DNA recognition and cleavage. The primers used to amplify the sgRNA-targeting regions for sequencing analysis are 5'- ACCCCCAAACAGAGATAACCC-3' + 5'-GCCTACACTACCTCCTAAAAGGG-3' (for OCLN), and 5'- AGGGCCAGGCTCCAAGTAG-3' + 5'- ACACGCCATGCCCGACT-3' (for CD81).

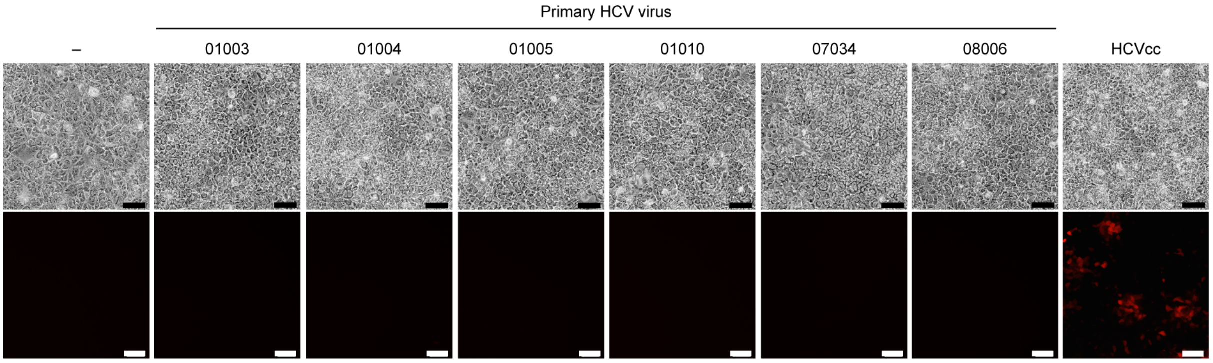

Supplementary Figure 8. The mCherry signal of the NlrD system in Huh7.5 cells in response to HCV infection. Total of 6 serum samples from different HCV patients were used at high MOI (Titer of viral samples was obtained by Abbott Realtime™ Test Assays), 01003 (genotype 2), 01004 (genotype 1b), 01005 (genotype 1b), 01010 (genotype 2), 07034 (genotype 3a) and 08006 (genotype 6a/6b). Cells were infected by virus in the presence of Dox (2 μg/ml) for 72 h before observation under microscope. Scale bar, 100 μm.
